# Synthesis of lithium oxy-thiophosphate solid electrolytes with Li_10_GeP_2_S_12_ structure by a liquid phase process using 2-propanol

**DOI:** 10.1039/d3ra03929c

**Published:** 2023-07-28

**Authors:** Shunichiro Shiba, Akira Miura, Yuta Fujii, Kiyoharu Tadanaga, Kota Terai, Futoshi Utsuno, Hiroyuki Higuchi

**Affiliations:** a Graduate School of Chemical Sciences and Engineering, Hokkaido University 060-8628 Hokkaido Japan; b Faculty of Engineering, Hokkaido University 060-8628 Hokkaido Japan tadanaga@eng.hokudai.ac.jp; c Lithium Battery Material Department, Advanced Materials Company, Idemitsu Kosan Company Limited Chiba 299-0293 Japan

## Abstract

Lithium oxy-thiophosphates isostructural with Li_10_GeP_2_S_12_ (LGPS) were synthesized by a liquid-phase process using 2-propanol as the solvent and Li_2_S and P_2_S_5_ as the starting materials. The XRD and ^31^P NMR results indicate that the synthesized compound has a slightly shrieked LGPS-type crystal structure where sulfur in PS_4_^3−^ is partially replaced by oxygen. The sample synthesized from the nominal composition of Li_2_S : P_2_S_5_ = 2.5 : 1 and at the annealing temperature of 300 °C exhibited the ionic conductivity of 1.6 × 10^−4^ S cm^−1^ at 25 °C. The synthesized solid electrolyte was found to be electrochemically stable in the potential range of 0–5 V, and also relatively stable under air with low relative humidity.

## Abstract 2

Crystal structure model of lithium oxy-thiophosphates (LPSO) prepared in the present study (a) and Li_10_GeP_2_S_12_ (b). Purple spheres indicate P, yellow spheres indicate S, red spheres indicate O, and green spheres indicate Ge.

## Graphical abstract

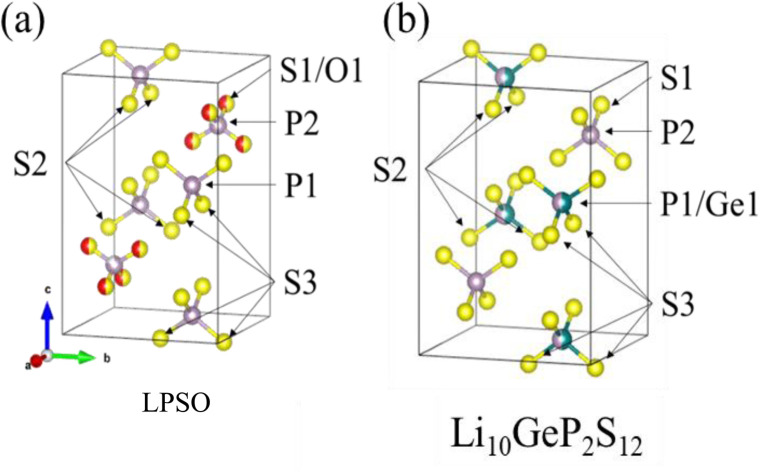

## 1. Introduction

In recent years, all-solid-state lithium secondary batteries have been actively studied as next-generation devices. In all-solid-state lithium secondary batteries, flame-retardant solid electrolytes have been used instead of flammable liquid electrolytes. Inorganic solid electrolytes are particularly safe at high temperatures owing to their low risk of ignition. In addition, solid electrolytes can be used over a wider range of temperatures because the ionic conductivity of solid electrolytes decreases less at low temperatures in comparison to liquid electrolytes.^1–3^

Among solid electrolytes, sulfide-based solid electrolytes are particularly promising owing to their high ionic conductivities, and easy processability stemming from their good mechanical properties and relative softness. Hence, they can be easily densified by pressing at room temperature to reduce the interfacial resistance:^4,5^ however, there is a problem that the reaction with moisture generates harmful hydrogen sulfide. In addition, electrochemical stability and stability against lithium metal are important properties required for solid electrolytes.

Various types of sulfide solid electrolytes have been reported. For example, various Li_2_S–P_2_S_5_ solid electrolytes can be synthesized by adjusting the composition of the raw materials, Li_2_S and P_2_S_5_.^6^ Li_7_P_3_S_11_,^7–9^ Li_2_S–P_2_S_5_ glass ceramics, and crystalline β-Li_3_PS_4_ (ref. 10 and 11) and Li_7_PS_6_ (ref. 12) exhibit ionic conductivities in the order of 10^−4^ S cm^−1^ or better at room temperature. Among solid electrolytes, Li_10_GeP_2_S_12_ (LGPS) exhibits particularly high ionic conductivity in the order of 10^−2^ S cm^−1^ at room temperature,^2^ which is comparable to that of liquid electrolytes. However, the problems with LGPS are that LGPS is unstable at both electrode/electrolyte interfaces. For example, a Li-depleted LGPS layer is formed at the cathode/electrolyte interface during charging, resulting in degradation, such as decreased capacity and increased resistance.^13^ LGPS is also susceptible to instability owing to the reduction of Ge cations in its structure by Li metals.^14^ Moreover, many solid electrolytes with the LGPS-type structure,^15,16^ including oxygen-containing compounds in the Li_2_S–P_2_S_5_ system (Li–P–S–O compounds) have been reported.^17,18^

There are two main processes for synthesizing sulfide solid electrolytes: solid-phase and liquid-phase processes. The liquid-phase process is more suitable for scaling up than the solid-phase process and is suitable for mass synthesis. In addition, the chemical interactions owing to the solvent may provide new reaction pathways that are different from those of the solid-phase process. Thereby, new solid electrolytes that cannot be synthesized with the solid-phase process are expected to be discovered.^19,20^

In this study, lithium oxy-thiophosphates isostructural with LGPS were prepared by a liquid-phase process using 2-propanol as the solvent and Li_2_S and P_2_S_5_ as the starting materials. Oxygen was introduced into the sulfide electrolyte in the Li_2_S–P_2_S_5_ system by the reaction of 2-propanol with the starting materials. The Rietveld analysis of the products obtained by this process showed that the obtained compound has an LGPS-type crystal structure, and that a part of the sulfur was selectively replaced by oxygen. Furthermore, the oxygen content surpassed that of most Li–P–S–O compounds reported so far prepared by solid-state reactions, including ball milling.^17,18^ The solid state ^31^P NMR spectra of the samples also supported the incorporation of oxygen into the structure.

## 2. Experimental

### 2.1. Synthesis of solid electrolytes

Li_2_S (Mitsuwa Chemical, 99.9%, <74 μm) and P_2_S_5_ (Aldrich, 99%) were mixed in an agate mortar for about 10 min. This mixed powder was added to super-dehydrated 2-propanol (Fujifilm Wako Pure Chemical, >99.7%), and the solution was stirred for 5 min. The obtained precursor solution was subsequently dried at 120 °C for 3 hours under a vacuum to remove the solvent. The obtained precursor powders were heat-treated at 300–500 °C for 2 hours with a heating rate of 2 °C min^−1^. The composition ratios of Li_2_S and P_2_S_5_ were in the range of 2 : 1–3.3 : 1. These processes were carried out in a dry Ar atmosphere.

### 2.2. Characterization

XRD measurements were performed under the following conditions to evaluate the crystal structure of the sample: CuKα with a tube voltage of 40 kV and current of 15 mA, a step of 0.02°, a scan angle of 2*θ* = 5–60°, and a scan speed of 2.5 deg min^−1^. The samples were sealed in a non-atmospheric holder. Si powder was used as the standard sample for angle correction. Rietveld analysis (Rigaku, SmartLab Studio II) was performed to determine the structural parameters using the measured XRD patterns.

Solid-state ^31^P magic-angle spinning (MAS) NMR spectra were obtained to investigate the structural units in the samples. The solid-state NMR measurement was performed under the following conditions: the rotational speed of the sample was 10 kHz, the pulse width was 90° pulse (1.3 μs) or 30° pulse (1.0 μs), the repetition time *d*_1_ was 30 s, and the number of accumulations was 64.

The ionic conductivity of the samples was evaluated *via* AC impedance spectra measurements. To prepare the cell for the measurements, 30 mg of the prepared sample was weighed, pressurized, and formed into a pellet of *φ* = 6 mm, and then the pellet was sandwiched between stainless steel current collectors to form blocking electrodes. The thicknesses of the pellets were 200–300 μm. The fabricated cell was sealed in a separable flask, and measurements were performed under Ar atmosphere at 25 °C (room temperature), 60 °C, and 90 °C. The measurement conditions were an applied voltage of 50 mV and a frequency of 7 × 10^6^ to 0.1 Hz.

Cyclic voltammetry was performed by sandwiching the pelletized sample between the working electrode (stainless steel) and the counter electrode/reference electrode (stainless steel laminated with metallic lithium foil). The cell was sealed in a separable flask, and measurements were performed under an Ar atmosphere. Three cycles were measured with a sweep rate of 1 mV s^−1^ and a potential range of −0.5–5 V *vs.* Li/Li^+^.

The stability of the solid electrolyte against moisture was estimated by measuring hydrogen sulfide generation. The powders of the solid electrolyte in a separable flask were exposed to an air with relative humidity of about 5%, and generated amount of H_2_S was estimated using an H_2_S gas sensor.

## 3. Results and discussion

**Fig. 1:**
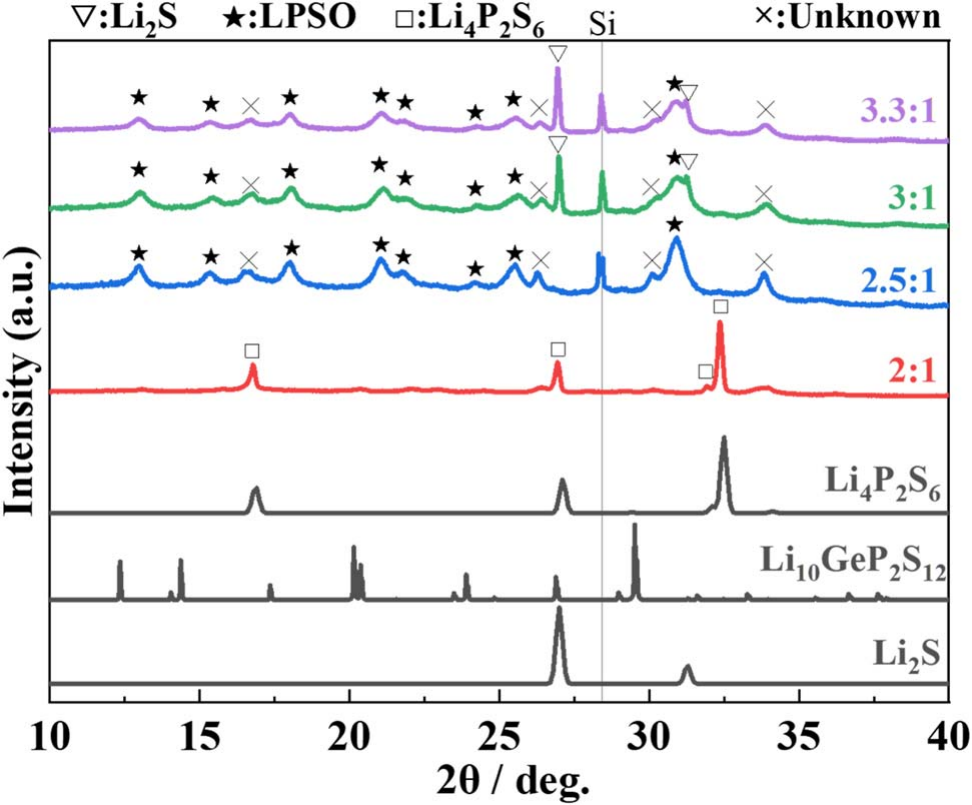
XRD patterns of the samples, which were prepared with the molar ratio of Li_2_S : P_2_S_5_ = 2 : 1, 2.5 : 1, 3 : 1, and 3.3 : 1, after heating at 400 °C. The indexed diffraction patterns of Li_2_S (ICSD: 60432), Li_10_GeP_2_S_12_ (ICSD: 241439) and Li_4_P_2_S_6_ (ICSD: 33506) are shown for comparison.

The XRD patterns of samples heat-treated at 400 °C of each composition ratio are shown in Fig. 1. In the sample with Li_2_S : P_2_S_5_ = 2 : 1, peaks of Li_4_P_2_S_6_ phase (marked with □) are mainly observed. In the samples with Li_2_S : P_2_S_5_ ratios ranging from 2.5 : 1 to 3.3 : 1, peaks that can be assigned to the LGPS-type structure (marked with ★)^2^ are appeared. The peak position and patterns are slightly different from those of the Li–P–S–O compounds reported thus far,^17,18^ but this phase was assumed to be a Li–P–S–O compound with an LGPS-type crystal structure because of the similarity of the patterns. Hereafter, this phase is referred to as “LPSO”. In addition to LPSO, peaks that cannot be assigned to a known phase (marked with x) were also observed. In the sample with Li_2_S : P_2_S_5_ = 3 : 1 and 3.3 : 1 samples, the peaks from residual Li_2_S (the raw material) were confirmed. However, in the sample with Li_2_S : P_2_S_5_ = 2.5 : 1, peaks of Li_2_S were not observed.

**Fig. 2:**
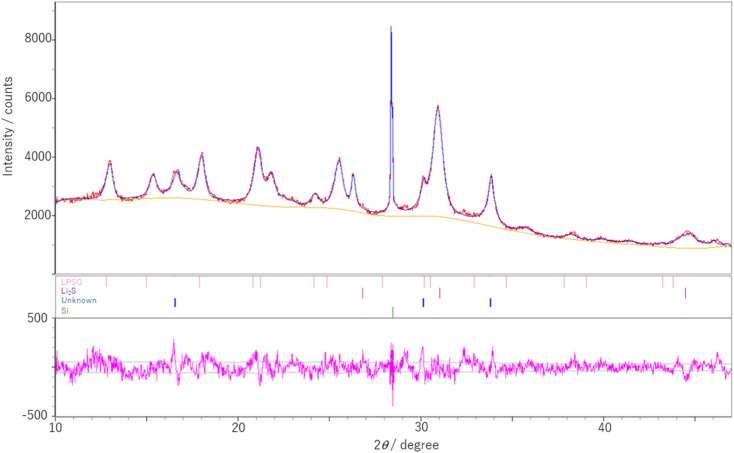
Rietveld profile of the prepared sample with the molar ratio of Li_2_S : P_2_S_5_ = 2.5 : 1 after heating at 400 °C. The red and blue lines show the measured and simulated data, respectively. The bars represent Bragg positions of each phase due to LPSO, Li_2_S, unknown and Si. The pink line is their residuals. In the LPSO phase, the occupancy of oxygen at S1/O1 sites is set to be half.

The results of the Rietveld analysis for the sample with Li_2_S : P_2_S_5_ = 2.5 : 1 are shown in Fig. 2. Refinement based on the structure of the LGPS-type Li–P–S–O electrolytes reported previously^17^ showed that LPSO has a space group *P*4_2_/*nmc* and lattice constants *a* = 8.1199(9) Å and *c* = 12.325(2) Å. These lattice constants were found to be smaller than those previously reported.^17,18^

**Fig. 3:**
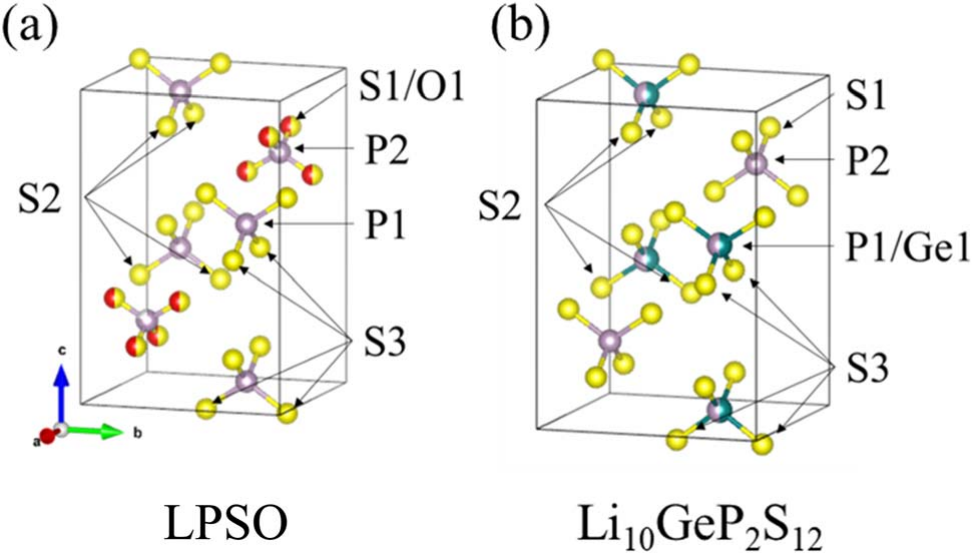
Crystal structure model of LPSO (a) and Li_10_GeP_2_S_12_ (b). Purple spheres indicate P, yellow spheres indicate S, red spheres indicate O, and green spheres indicate Ge. This image is produced by VESTA.^22^

**Fig. 4:**
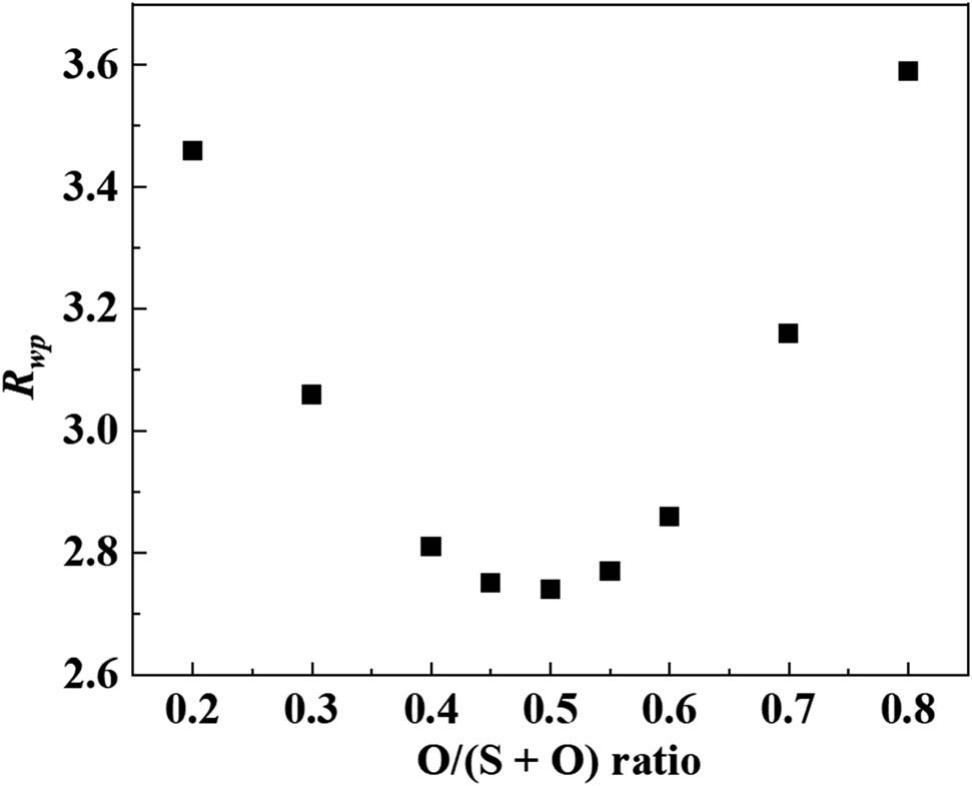
*R*_wp_ for O occupancy at S1/O1 sites. A smaller value of *R*_wp_ on the vertical axis indicates a better fitting.

A model of the crystal structure obtained from the refinement results is shown in Fig. 3(a), where only the P(S,O)_4_ unit is represented and Li is not shown. The crystal structure model indicated that there is a partial defect at the P2 site. Although the occupancies of the S2 site were unit, the occupancies of the S1 site does not reach unit. The fitting of the refinement by changing the occupancies of S and O at the S1 site is shown in Fig. 4. A smaller value of *R*_wp_ on the vertical axis indicates a better fitting. Fig. 4 shows that the best fitting was obtained when the occupancy of O was 0.5, that is, when the S/O ratio was 50 : 50. The expected composition of LPSO from these results is calculated to be Li_10_P_2.8_S_10_O_2_. The amount of lithium was determined by considering charge compensation. This composition has a larger proportion of O than the previously reported LGPS-type Li–P–S–O compound.^17,18^ Since the ionic radius of O is smaller than that of S, the lattice constants of the present LPSO are small. The difference in the composition ratio from the starting materials can be attributed to the presence of unknown or amorphous phases and/or the vaporization of the phosphorus and sulfur components during the drying process at 120 °C for 3 h under a vacuum. These vaporizations may be attributed to HS(S)P(OC_3_H_7_)_2_ with low boiling point (72–74 °C under 0.7 Torr), which can be produced by the reaction of P_2_S_5_ and 2-propanol.^21^

The substitution of only the S1 site can be explained by comparing it with the structure of Li_10_GeP_2_S_12_ shown in Fig. 3(b). The obtained LPSO with the LGPS-type structure has PS_4_ units at the P1 site and P(S/O)_4_ units at the P2 site, and their size relationship is PS_4_ > P(S/O)_4_ because of the difference in the ionic radii of S and O. On the other hand, Li_10_GeP_2_S_12_ has (P/Ge)S_4_ units at the P1 site and PS_4_ units at the P2 site, and their size relationship is (P/Ge)S_4_ > PS_4_, owing to the difference in the ionic radii of P and Ge. Therefore, only the S1 site was considered to be substituted because the position and size relationship of these units are consistent with those of Li_10_GeP_2_S_12_, resulting in a stable structure.

**Fig. 5:**
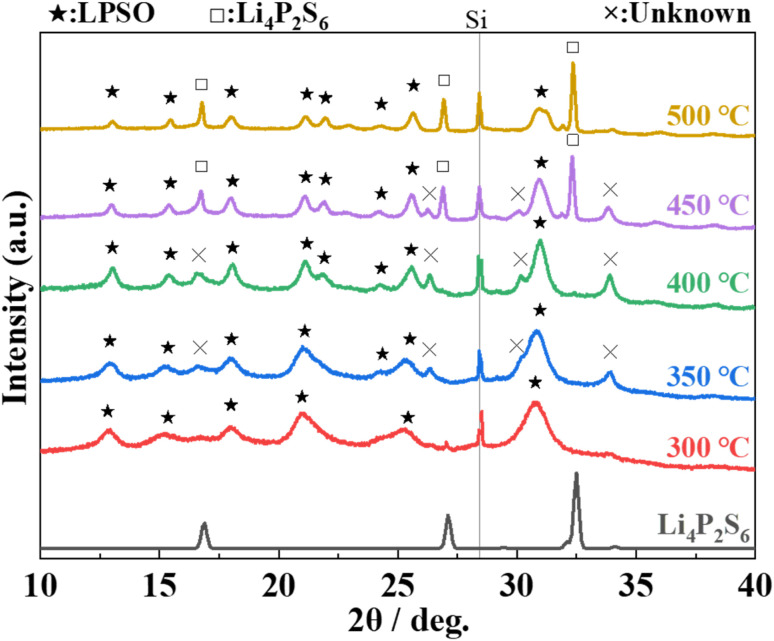
XRD patterns of the prepared samples with the molar ratio of Li_2_S : P_2_S_5_ = 2.5 : 1 after heating at 300–500 °C. The indexed diffraction pattern of Li_4_P_2_S_6_ (ICSD: 33506) is shown for comparison.

The XRD patterns of the samples heat-treated at various temperatures with Li_2_S : P_2_S_5_ = 2.5 : 1 are shown in Fig. 5. The peaks assigned to Li_4_P_2_S_6_ appeared and the intensity of the peak increased with an increase in the heat-treatment temperature from 400 °C to 500 °C. The intensity of the peaks based on the unknown phase is rather small in the sample heat-treated at 300 °C. These results indicate that heat treatment at 300 °C does not produce by-products, and that the obtained phase is almost a single phase of LPSO. However, because the full width at half maximum (FWHM) of the peaks was relatively large and a halo was also observed as the background, an amorphous phase was considered to co-exist. Considering the difference between the composition ratio (Li_2_S : P_2_S_5_ = 2.5 : 1) and the composition of LPSO as described above (Li_10_P_2.8_S_10_O_2_), this amorphous phase is considered to contain a smaller Li/P ratio than LPSO.

**Fig. 6:**
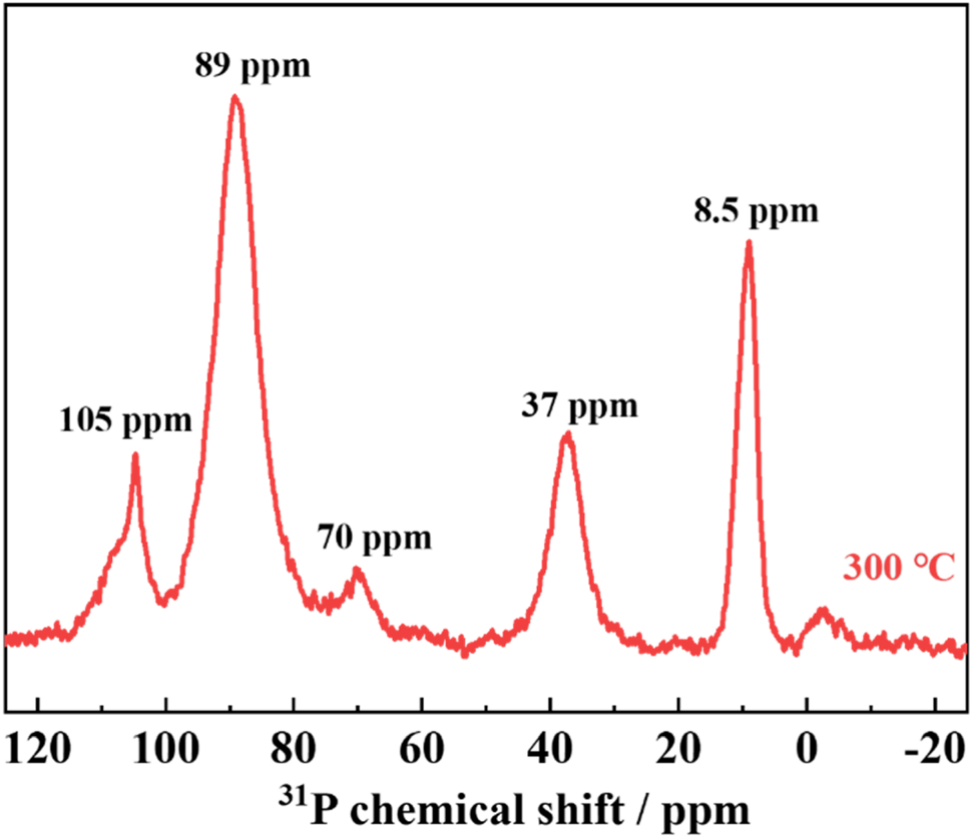
Solid-state ^31^P MAS NMR spectra of samples heat-treated at 300 °C with Li_2_S : P_2_S_5_ = 2.5 : 1.

The NMR spectra of the samples annealed at 300 °C are shown in Fig. 6. The peak at around 89 ppm is attributed to PS_4_^3−^ or PS_3_O^3−^, the peak at around 69 ppm to PS_2_O_2_^3−^, the peak at around 37 ppm to PSO_3_^3−^, and the peak at around 8.5 ppm to PO_4_^3−^.^23–25^ The NMR results also confirm that LPSO has structural units in which a part of the S in PS_4_^3−^ is replaced by O. The peak at approximately 105 ppm is attributed to P_2_S_6_^4−^,^25^ which may indicate the presence of P_2_S_6_^4−^ in the unknown and/or amorphous phases.

**Fig. 7:**
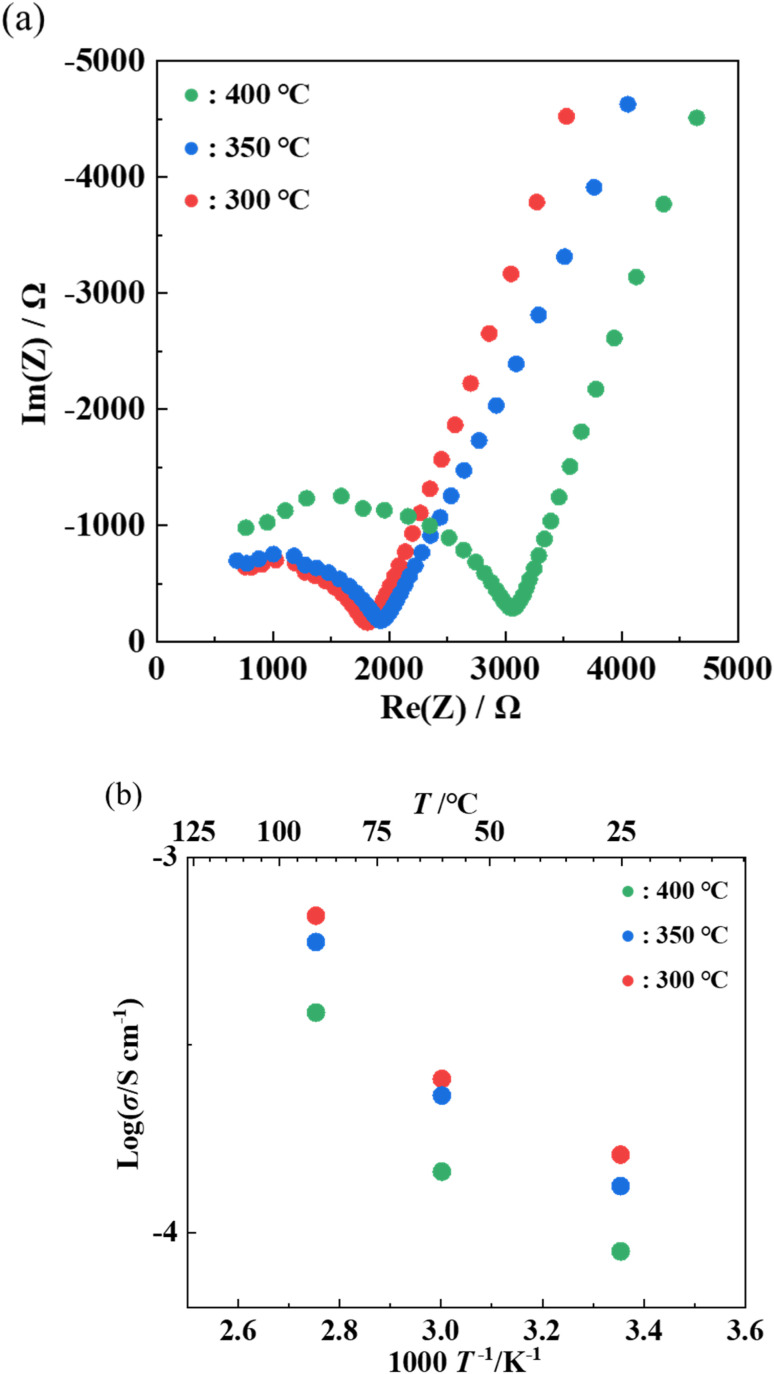
Nyquist plots at room temperature(a) and Arrhenius plots (b) of samples heat-treated at 300 °C, 350 °C, or 400 °C with Li_2_S : P_2_S_5_ = 2.5 : 1.

AC impedance measurements were performed on each of the samples heat-treated at 300–400 °C to measure ionic conductivity. Fig. 7(a) and (b) shows the Nyquist plots and the Arrhenius plots of the samples, respectively. The ionic conductivity at 25 °C was 8.9 × 10^−5^ S cm^−1^ with annealing at 400 °C, 1.3 × 10^−4^ S cm^−1^ with annealing at 350 °C, and 1.6 × 10^−4^ S cm^−1^ with annealing at 300 °C, respectively. The activation energy was 20 kJ mol^−1^ for all samples. The sample annealed at 300 °C exhibited the highest conductivity among the previously reported LGPS-type Li–P–S–O compounds.^17,18^ The decrease in the ionic conductivity with higher annealing temperatures can be attributed to the formation of by-products with the heat treatment at higher temperatures, as can be seen from the XRD pattern in Fig. 5.

**Fig. 8:**
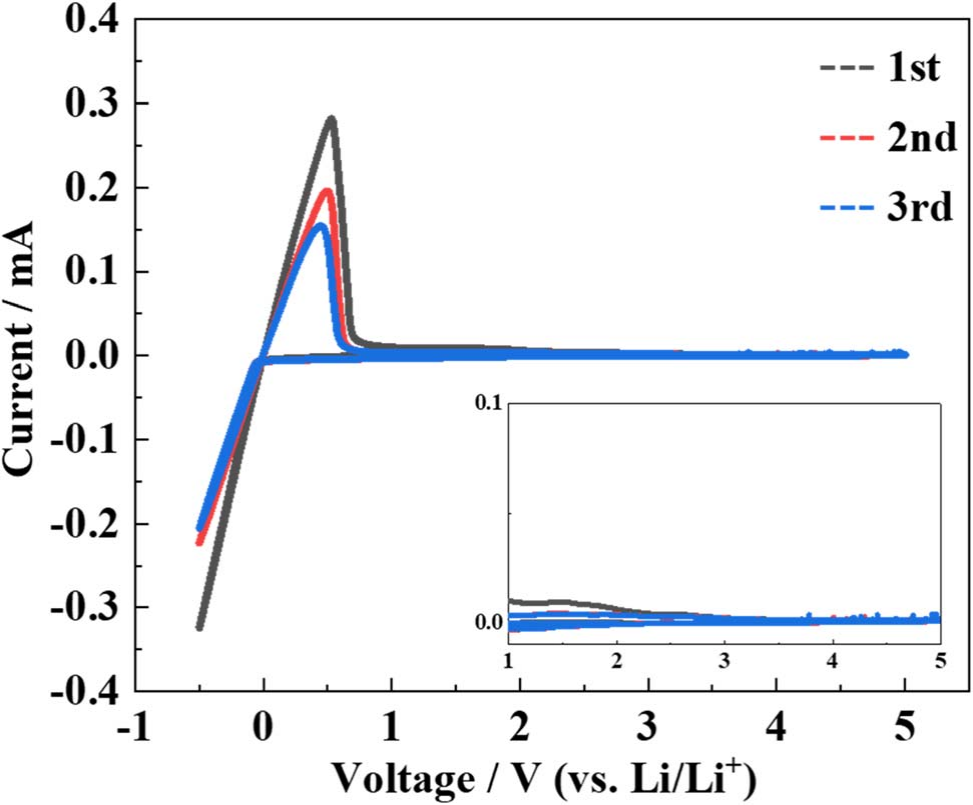
Cyclic voltammograms of samples heat-treated at 300 °C with Li_2_S : P_2_S_5_ = 2.5 : 1. The inset shows a magnified view of current values from 1 to 5 V.

Cyclic voltammetry measurements were performed to evaluate the electrochemical stability of the LPSO as a solid electrolyte. The cyclic voltammograms are shown in Fig. 8. No distinct current peaks other than the lithium redox at approximately 0 V were observed. This indicates that LPSO has a wide potential window between the potential range of −0.5 to 5.0 V and is stable to metallic lithium. As mentioned earlier, LGPS is electrochemically unstable because of Ge cation reduction and also the reduction of the PS_4_ unit to form Li_3_P. However, the LPSO exhibits a wide electrochemical window. The wide electrochemical window should be because LPSO does not contain easily reduced Ge components, and the introduction of oxygen to the structural units should contribute to the stabilization. The stabilization by oxygen incorporation may occur because the P–O bond is more energetically stable than the P–S bond.

Hydrogen sulfide generation was measured in an air with low relative humidity to evaluate the stability of the sample. The amount of hydrogen sulfide generation per gram of sample exposed to the air with relative humidity of approximately 5% for 3 h is listed in Table 1. For comparison, β-Li_3_PS_4_, a typical Li_2_S–P_2_S_5_ solid electrolyte prepared by a solution process,^26^ was also measured. As shown in Table 1, the amount of hydrogen sulfide generation in LPSO after 3 h of exposure to the air was approximately 1/3 of that in β-Li_3_PS_4_. Thus, LPSO shows superior stability against moisture than β-Li_3_PS_4_ consisting only of PS_4_ units, because of the introduction of O. Because approximately 1/6 of the total S was only substituted by O, the three-fold change in the amount of generation is not due to the decrease in S in the structural units, but due to the higher stability of the PS_4−*x*_O_*x*_ units by the introduction of O.

**Table 1:** Hydrogen sulfide generation per gram of sample after 3 h of exposure to air with relative humidity of about 5%

	Amount of hydrogen sulfide generated after 3 h (cm^3^ g^−1^)
β-Li_3_PS_4_	1.6
LPSO	0.5

The most novel feature of this study is that the lithium oxy-thiophosphate solid electrolyte was synthesized using a liquid-phase process. Liquid-phase synthesis is a useful process for the synthesis of solid electrolytes because of its short synthesis time and scale-up capability.^16^ Moreover, the composition of the crystalline phase is richer in oxygen than that synthesized by the solid-state reaction. Thus, the results obtained in this study indicate that lithium oxy-thiophosphate solid electrolytes synthesized by the liquid-phase process are promising as solid electrolytes for all-solid-state lithium secondary batteries.

## 4. Conclusion

Novel lithium oxy-thiophosphate solid electrolytes with the LGPS-type structure were synthesized, where approximately a quarter of sulfur content was replaced by oxygen (LPSO) *via* a liquid-phase process using 2-propanol. The XRD and ^31^P NMR results indicated that LPSO contained structural units where a part of sulfur in PS_4_^3−^ was selectively replaced by oxygen. Under the composition ratio of Li_2_S : P_2_S_5_ = 2.5 : 1 and annealing temperature of 300 °C, the obtained sample exhibited the ionic conductivity of 1.6 × 10^−4^ S cm^−1^ at 25 °C. The solid electrolyte was electrochemically stable in the potential range of 0–5 V, and relatively stable under air with low relative humidity. The lithium oxy-thiophosphate solid electrolytes synthesized by a liquid-phase process in this study are promising as solid electrolytes for all-solid-state lithium secondary batteries.

## Conflicts of interest

There are no conflicts to declare.

## Supplementary Material
